# Unveiling the Role of Rumen Microbiome in Modulating Intramuscular Fat Deposition of Pingliang Red Cattle

**DOI:** 10.1002/fsn3.71681

**Published:** 2026-04-15

**Authors:** Hong Meng, Shuanping Zhao, Hai Jin, Huibin Zhang, Qian Li, Lupei Zhang, Junwei Hu, Fanyun Kong, Xinyi Du, Qinggang Li, Muhammad Ajwad Rahim, Lei Xu, Yanfeng Xue

**Affiliations:** ^1^ College of Animal Science and Technology Anhui Agricultural University Hefei China; ^2^ Anhui Provincial Key Laboratory of Livestock and Poultry Product Safety, Institute of Animal Husbandry and Veterinary Medicine Anhui Academy of Agricultural Sciences Hefei China; ^3^ State Key Laboratory of Animal Biotech Breeding, Institute of Animal Sciences Chinese Academy of Agricultural Sciences (CAAS) Beijing China; ^4^ Academy of Pingliang Red Cattle Pingliang China; ^5^ Jingchuan County Animal Husbandry and Veterinary Center Pingliang China

**Keywords:** intramuscular fat deposition (IMF), meat quality, metagenomics, microbiome, Pingliang red cattle, rumen

## Abstract

Pingliang Red cattle is renowned for its tender meat and symmetrical intramuscular fat (IMF) deposition. Rumen microbiota are crucial for energy metabolism and nutrient acquisition in cattle, significantly influencing IMF deposition. Therefore, this study aimed to explore how rumen microbiota impact IMF deposition in Pingliang Red cattle. 34 castrated Pingliang Red cattle were subjected to the same management for 2 months, followed by centralized and unified slaughtering. Based on the measured IMF content in the longissimus dorsi, 18 cattle were selected and divided into a high‐intramuscular‐fat group (HIMF, *n* = 9) and a low‐intramuscular‐fat group (LIMF, *n* = 9). Rumen fluid was subsequently collected for metagenomic sequencing. Results showed significant differences in taxonomic abundance at both the genus and species levels, the relative abundance of carbohydrate‐active enzyme (CAZy) families, and functional profiles (*p* < 0.05). Specific rumen microbes, such as *Limosilactobacillus panis* (AUC = 0.765) and *Fibrobacter succinogenes* (AUC = 0.753), served as potential biomarkers for HIMF deposition in Pingliang Red cattle. With the exception of *Bacillus*, *Fibrobacter succinogenes, Limosilactobacillus panis*, *Prevotella intermedia* , and *Streptomyces* exhibited positive correlations with IMF content. Functional analysis based on KEGG orthology (KO) indicated that specific enzymes promote IMF deposition by regulating the metabolism of short‐chain fatty acids (SCFAs), long‐chain fatty acids (LCFAs), and lipopolysaccharides, as well as insulin signaling. These findings provide a theoretical reference for regulating rumen microbial communities to improve IMF deposition.

## Graphical abstract

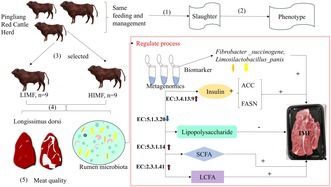

**Abbreviations**
Ala: alanine
Arg: arginine
Asp: aspartate
AUC: area under the curve
CAzy: carbohydrate‐active enzymes
Cys: cysteine
Glu: glutamate
Gly: glycine
HIMF: high intramuscular fat
His: histidine
Ile: isoleucine
IMF: intramuscular fat
KO: KEGG orthology
LCFA: long chain fatty acid
Leu: leucine
LIMF: low intramuscular fat
Lys: lysine
Met: methionine
Phe: phenylalanine
Pro: proline
ROC: receiver operating characteristic
SCFA: short chain fatty acid
Ser: serine
Thr: threonine
Tyr: tyrosine
Val: valine

## 1 Introduction

The Pingliang Red cattle are regionally adapted to the specific environmental conditions of Pingliang city, China (Wang et al. 2024). By the end of 2024, the population of Pingliang Red cattle had reached 350,000 with 144,000 slaughtered annually. Pingliang Red beef exhibits excellent meat texture with abundant and evenly distributed intramuscular fat deposits. Compared with beef from other breeds, Pingliang Red beef contains 11.58% and 11.06% higher levels of oleic acid and linoleic acid, respectively. The ratio of polyunsaturated to saturated fatty acids (P:S) is 0.13. The total content of amino acids derived from Strecker degradation, key aroma precursors, accounts for 3.96% of total amino acids. According to evaluations by authoritative experts, high‐grade marbled beef from Pingliang Red cattle meets the national standard for premium beef in China.

In recent years, research has revealed that ruminants with high IMF exhibit distinct rumen microbiota profiles compared to their low‐IMF counterparts (Shi et al. 2024), suggesting that modulation of rumen microbiota may serve as a potential strategy to enhance IMF deposition in beef cattle. Using 16S rRNA gene sequencing, Kim et al. (2020) investigated the rumen microbiota of 14 Hanwoo beef cattle and identified that the *RFP12, Verrucobacteria, Spirochetes, Porphyromonas*, and *Paludibacter* were more abundant in the high‐marbling group, whereas *Olsenella* was prevalent in the low‐marbling group (Kim et al. 2020). That study demonstrated that Hanwoo cattle with divergent IMF contents showed distinct rumen microbial composition, diversity, and inferred metabolic characteristics, which may contribute to beef marbling development. Similar findings regarding microbiota‐IMF associations have been reported in studies on yak (Xiong et al. 2024) and Tibetan sheep (Sha et al. 2023).

However, the specific microbial taxa and functional pathways linking rumen microbial dynamics to IMF accumulation remain poorly characterized, primarily due to limitations of traditional culture‐dependent methods that fail to capture more than 90% of unculturable rumen microbes (Urga et al. 2025). Metagenomic technology addresses this gap by enabling direct, cultivation‐independent sequencing of total rumen DNA, providing a holistic view of microbial taxonomic diversity and functional gene repertoires. Such approaches have been extensively applied to investigate the composition and diversity of gastrointestinal microbiota and their impact on IMF deposition. For example, Yang, Bao, et al. (2024) utilized metagenomic sequencing to demonstrate that dietary niacin supplementation in Xiangzhong Black cattle increased the abundance of specific rumen bacteria, including *Ligulactobacillus ruminis*, *Anaerovibrio lipolyticus*, and *Mitsuokella multacida*, which were positively correlated with IMF content. Similarly, metagenomic sequencing combined with targeted metabolomics has revealed that the intestinal microbiota of Angus cattle can regulate meat quality. Zheng et al. (2022) reported that the expression of host genes related to beef quality—*ATP2A1, MSTN, ACTN3, MYLPF, MYL1*, and *TNNT3*, were positively correlated with the abundance of specific intestinal bacteria, such as *Bacteroides uniformis* , *Bacteroides vulgatus* and *Ruminococcus inulivorans*. Therefore, the present study employed metagenomics to dissect rumen microbe‐IMF associations, aiming to clarify microbial regulatory mechanisms and provide a theoretical basis for beef quality improvement.

## 2 Material and Methods

### 2.1 Ethics Certification

In this study, all the experimental procedures were approved by the Experimental Animal Management Committee (EAMC) of the Pingliang Red Cattle Research Institute and the Animal Care and Use Committee of the Anhui Academy of Agricultural Sciences (approval number: A22‐CS15). The study was conducted in accordance with the *National Standard of Laboratory Animals Guidelines for Ethical Review of Animal Welfare* (GB/T 35892‐2018) and the *Guide for the Care and Use of Laboratory Animals: Eighth Edition*.

### 2.2 Animal Materials

In this study, 34 castrated Pingliang Red cattle aged 34 months were maintained under uniform feeding and management practices at Xukang Food Co. Ltd., Pingliang City, Gansu Province, China. An adaptation phase lasted 10 days, followed by a 50‐day experimental period. Diets were formulated according to NRC (2007) guidelines, with nutrient composition and forage proportions presented in Table 1. The cattle were slaughtered at 36 months of age at a centralized abattoir, where body size traits were measured prior to slaughter. After slaughter, the longissimus dorsi muscle was excised from each animal; marbling scores were assessed immediately, while IMF content was determined in the laboratory within 24 h. Rumen fluid and serum samples were collected immediately post‐slaughter.

**TABLE 1:** Ingredients and nutrients composition of basal diets in Pingliang red cattle.

Forage	Proportion%	Unit	Nutrient levels
Rolled corn	39.81	ME, MJ/kg	11
Corn silage	22	CP%	11.89
Straw	18	EE%	3.91
Soybean meal	11.1	NDF%	30.68
Wheat bran	5	ADF%	17.06
Dust‐proof soft straw hay	2.21	Ca %	0.5
Calcium hydrogen phosphate	0.28	P%	0.26
Premix^a^	1.6		

^a^ Premix (per kg) cotained: Vitamin A 15 mg, Vitamin D 0.15 mg, Vitamin E 100 mg, Vitamin K 8 mg, Vitamin B1 6mg, Vitamin B2 45mg, Vitamin B6 40mg, Vitamin C 75 mg, Nicotinic acid 65 mg, Cu 5 mg, Fe 65 mg, Zn 73 mg, Mn 40 mg, I 0.8 mg, Se 0.6 mg, Cr 0.6 mg.

### 2.3 Determination of IMF Content and Grouping

The marbling score was assessed by visually comparing the cross‐section of the longissimus dorsi muscle obtained between the 12th and 13th ribs against a standard reference card. According to the Chinese Beef Marbling Standard, marbling scores were categorized into five grades (1–5), with higher grades indicating more pronounced marbling and greater IMF content. The IMF content of the meat samples was determined by Soxhlet extraction (Chen, Wu, et al. 2022). Subsequently, 9 cattle with the highest IMF content (9.2%–16.6%) and 9 with the lowest (2.4%–5.5%) were selected and designated as the high‐intramuscular‐fat (HIMF) group (*n* = 9) and the low‐intramuscular‐fat (LIMF) group (*n* = 9), respectively.

### 2.4 Meat Quality Measurement

The loin eye area (LEA) was measured at the 12th rib interface by tracing the longissimus dorsi outline onto acetate paper and calculated using the dot grid method. Muscle *p*H was measured at 24 h postmortem using a calibrated HANNA HI99163 pH meter (Hanna Instruments). Shear force was determined according to Hou et al. (2014) using a Warner‐Bratzler blade attached to a TA‐XT Plus texture analyzer. Cooking loss was calculated after water‐bath cooking to 70°C internal temperature. WHC was determined by the centrifugation method (1000 × g, 5 min). Crude protein and moisture contents were determined by the Kjeldahl method (GB 5009.5‐2016) and direct drying (GB 5009.3‐2016), respectively. All measurements were performed in triplicate.

### 2.5 Amino Acids and Fatty Acids Measurement

Amino acid was determined according to GB 5009.124‐2016 National Food Safety Standard ‘Determination of amino acid in food’, using an amino acid analyzer, with a ≥ 0.99 linearity of amino acid determination, 98%–102% recovery rate, and ≤ 6% relative standard deviation. Fatty acid composition was determined using chloroform‐methanol (2:1, v/v) according to Folch et al. (1957). After saponification and methylation, fatty acid methyl esters (FAMEs) were analyzed by gas chromatography (Thermo Trace 1300) equipped with a flame ionization detector and a TG‐FAME capillary column (50 m × 0.25 mm × 0.20 μm). Helium was used as the carrier gas at 0.63 mL/min. The injector and detector temperatures were 250°C and 300°C, respectively. The column temperature was programmed from 80°C (1 min) to 160°C (20°C/min, 1.5 min), to 196°C (3°C/min, 8.5 min), and finally to 250°C (20°C/min, 3 min) (Beccaria et al. 2018; Hoving et al. 2018). Samples (1 μL) were injected with a split ratio of 1:20. Fatty acids were identified by comparing retention times with a 37‐component FAME standard (Supelco) and quantified using an internal standard method.

### 2.6 Rumen Volatile Fatty Acids (VFAs) Content and Serum Biochemical Indices Measurement

Rumen fluid VFAs were analyzed by gas chromatography (GC) using a modified method from Qin (1968). Briefly, 1 mL of rumen fluid was mixed with 0.2 mL phosphoric acid‐butyric acid solution (containing crotonic acid as the internal standard) and stored at −20°C overnight. After thawing, the mixture was centrifuged at 12,000 rpm for 10 min. The supernatant was filtered through a 0.22 μm membrane and diluted 5‐fold with double‐distilled water. The filtrate (1 μL) was injected into a Shimadzu GC‐9A gas chromatograph (Shimadzu Corp., Kyoto, Japan) equipped with a flame ionization detector (FID) and a CP‐WAX capillary column (30 m × 0.53 mm ID×1.0 μm). Nitrogen was used as the carrier gas; injector and detector temperatures were 200°C, and the column temperature program was 100 to 150°C at 3°C/min. VFA concentrations were quantified using crotonic acid as internal standard.

Total cholesterol (TC) was measured using a Leadman RT‐6100 Microplate Reader. Non‐esterified fatty acids (NEFA), triglycerides (TG), LDL, HDL, and glucose (GLU) were assayed using ELISA kits (Nanjing Jiancheng Bioengineering Institute, China). Serum insulin (INS) and IGF‐1 were quantified using ELISA kits (Quanzhou Ruixin Biotechnology, China). All assays were performed according to the manufacturer's protocols.

### 2.7 Metagenomics Sequencing

Genomic DNA was extracted from rumen samples utilizing the MagPure Stool DNA KF Kit B (MAGEN, Guangzhou, China) following the manufacturer's instructions. DNA concentration and integrity were assessed using a NanoDrop spectrophotometer and agarose gel electrophoresis (0.8%). Subsequently, a DNA library was constructed with the BGI Optimal DNA Library Prep Kit (BGI‐Shenzhen, China). After circularization and rolling circle amplification, high‐quality DNA nanoballs (DNBs) were sequenced on the DNBSEQ‐T7 platform (BGI).

### 2.8 Bioinformatic Analysis

Adaptor sequences and low‐quality reads were filtered using SOAPnuke v.2.2.1 (Chen et al. 2018). Reads containing > 0.1% ambiguous bases (N), > 50% low‐quality bases (*Q* < 20), or adaptor sequences were discarded. Clean reads were aligned to the bovine reference genome (ARS‐UCD1.2) using Bowtie 2 (2.4.4) to remove host contamination (Langmead and Salzberg 2012). Host‐free reads were assembled de novo using MEGAHIT (v1.1.3) (Li et al. 2015), and contigs < 300 bp were removed.

Gene prediction was performed using MetaGeneMark (Zhu et al. 2010), and redundancy was reduced using CD‐HIT (95% identity, 90% coverage) (Fu et al. 2012). Gene abundance was quantified as TPM (Transcripts Per Million) using Salmon (Patro et al. 2017). Non‐redundant genes were functionally annotated using DIAMOND v2.0.8 (Buchfink et al. 2014) with BLASTP (*E*‐value ≤ 1e−5) against CAZy (v102) (Lombard et al. 2014; Drula et al. 2022), eggNOG (v5.0) (Huerta‐Cepas et al. 2019), COG (20201125) (Galperin et al. 2015), and KEGG (v101) databases (Kanehisa and Goto 2000). Taxonomic annotation was conducted using Kraken (Wood et al. 2019) LCA algorithm using the Nt (202011) database. Differentially abundant phyla, genera, and KOs were identified using the Wilcoxon rank‐sum test (Matsouaka et al. 2016).

### 2.9 Statistical Analysis

The α‐diversity indices (observed species, Chao1, Shannon, and Simpson) were calculated using R packages (v4.1.2). Principal coordinate analysis (β‐diversity) was assessed by PCoA based on Euclidean, Bray‐Curtis (Bray and Curtis 1957), and Jensen‐Shannon Divergence (JSD) distances (Majtey et al. 2005). Differences in α‐ and β‐diversity between HIMF and LIMF groups were tested using Wilcoxon rank‐sum test. Differential microbial taxa and carbohydrate‐active enzymes (CAZymes) were identified using LEfSe (|LDA| > 2.0) and DESeq2 (*p* < 0.05), respectively. Visualizations were generated using ggpubr and pheatmap.

Microbial abundance data were log_10_(x + 1) transformed prior to correlation analysis; other indicators used raw data. Heatmaps were generated using the BioDeep Platform (https://www.biodeep.cn). Phenotypic traits, meat quality, and rumen VFAs were analyzed using Student's *t*‐test, while microbial abundances at genus and species levels were analyzed by independent *t*‐test. ROC analysis was performed using IBM SPSS Statistics 25.0.

## 3 Results

### 3.1 The Meat Quality of Pingliang Red Cattle

Slaughter performance and meat quality of castrated Pingliang Red cattle are presented in Table 2. No significant differences were observed in slaughter traits between HIMF and LIMF groups (*p* > 0.05). As shown in Table 3, the HIMF group exhibited significantly higher IMF% marbling score and cooking yield, but lower moisture, crude protein, and shear force than the LIMF group (*p* < 0.05).

**TABLE 2:** Phenotypic characteristics of Pingliang red cattle.

	LIMF (*n* = 9) mean ± SEM	HIMF (*n* = 9) mean ± SEM	*p*
Pre‐slaughter weight (kg)^a^	645.667 ± 27.837	629.778 ± 22.754	0.229
Hot carcass weight (HCW) (kg)^a^	380.644 ± 15.627	365.533 ± 18.769	0.099
Dressing %	58.964 ± 0.760	58.020 ± 1.409	0.115
Loin eye area (cm^2^)	87.222 ± 7.510	80.667 ± 8.589	0.124
Backfat thickness (cm)	1.956 ± 0.546	2.433 ± 0.834	0.194

^a^ Sample size may limit the generalizability of these findings.

**TABLE 3:** The meat quality of Pingliang red cattle.

	LIMF (*n* = 9) mean ± SEM	HIMF (*n* = 9) mean ± SEM	*p*
IMF%	3.867 ± 0.921	12.744 ± 2.402^a^	< 0.01
Marbling score	2.778 ± 0.416	4.667 ± 0.471^a^	< 0.01
Water content (%)	71.111 ± 1.149	64.322 ± 2.377^a^	< 0.01
Crude protein(%)	23.100 ± 0.452	20.756 ± 0.751^a^	< 0.01
Shear force(kgf)	5.740 ± 1.838	3.992 ± 0.823^a^	< 0.01
Water‐Holding Capacity%	31.609 ± 3.300	29.190 ± 1.831	0.089
*p*H(24 h)	5.32 ± 0.489	5.52 ± 0.045	0.265
Cooking‐loss(%)	22.72 ± 1.771	20.26 ± 2.084^a^	< 0.05

^a^ Values within a row with different superscripts differ significantly at *p* < 0.05.

### 3.2 Amino Acids, Fatty Acids and VFAs Content

**FIGURE 1:**
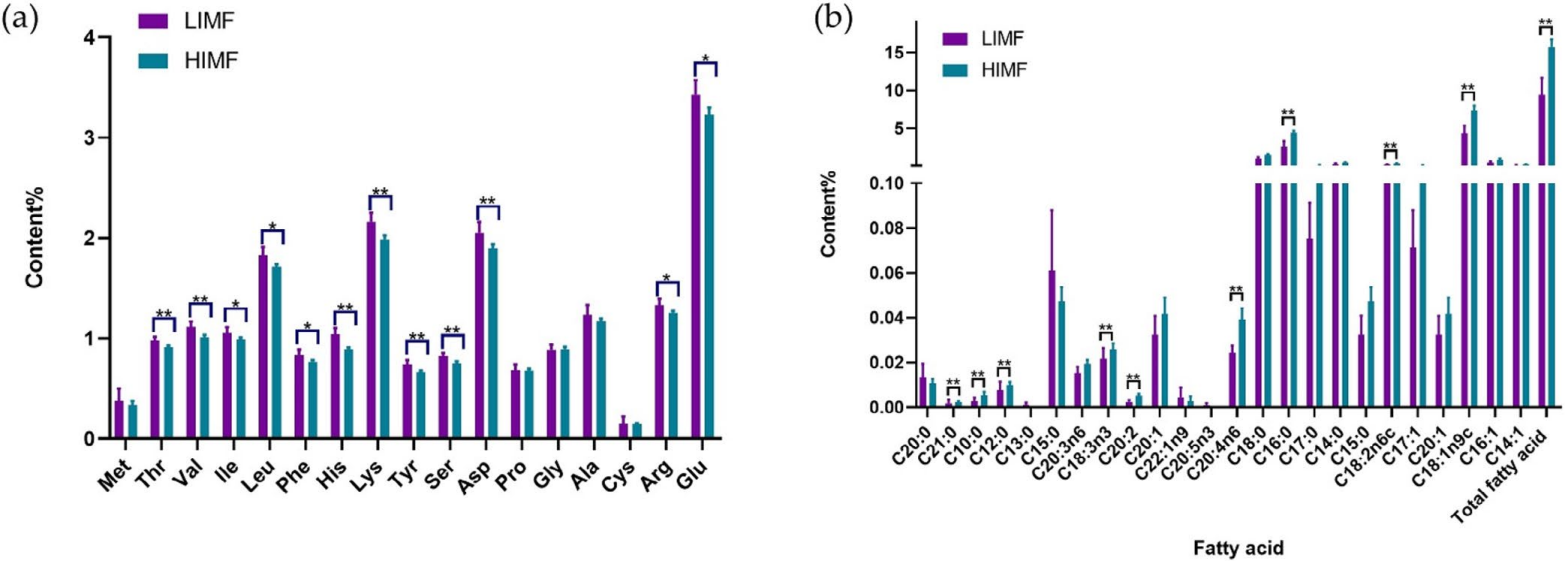
Information of amino acids and fatty acids in HIMF and LIMF groups. Values within a row with different superscripts differ significantly at *p < 0.05, **p < 0.01.

Concentrations of 12 amino acids were significantly lower in the HIMF group (*p* < 0.05; Figure 1a). Additionally, total fatty acids and 9 individual fatty acids (C10:0, C12:0, C16:0, C18:1n9c, C18:2n6c, C18:3n3, C20:2, C20:4n6, C21:0) were significantly more abundant in the HIMF group (*p* < 0.05; Figure 1b). As shown in Table 4, acetate, propionate and TVFAs were significantly increased in the HIMF group, resulting in elevated (*p* < 0.05), whereas butyrate and valerate were significantly decreased (*p* < 0.05). In serum, TC, NEFA, TG, INS, IGF‐1, and LDL were significantly higher in HIMF than in LIMF (*p* < 0.05), while HDL and GLU were significantly lower (*p* < 0.05).

**TABLE 4:** Differences in ruminal VFA content and serum biochemical indices.

VFA	LIMF	HIMF	*p*
Acetate(μmol/g)	56.065 ± 5.485	60.886 ± 2.889^a^	0.045
Propionate(μmol/g)	9.156 ± 0.884	10.184 ± 1.015^a^	0.046
Isobutyrate(μmol/g)	3.130 ± 0.301	2.857 ± 0.250	0.065
Butyrate(μmol/g)	7.821 ± 0.708	8.645 ± 0.835^a^	0.049
Isovalerate(μmol/g)	1.402 ± 0.135	1.536 ± 0.136	0.064
Valerate(μmol/g)	0.192 ± 0.014	0.177 ± 0.015^a^	0.048
Total VFAs (μmol/g)	77.751 ± 5.956	84.300 ± 3.203^a^	0.015
*Serum biochemical indices*			
TC (mmol/L)	7.938 ± 1.343	9.337 ± 0.911^a^	0.027
TG (mmol/L)	0.462 ± 0.052	0.536 ± 0.055^a^	0.014
GLU (mmol/L)	8.511 ± 1.801	6.392 ± 1.987^a^	0.040
NEFA (μmol/L)	937.077 ± 64.642	1182.503 ± 240.227^a^	0.013
INS(ng/mL)	1.307 ± 0.275	1.855 ± 0.598^a^	0.031
IGF‐1(ng/mL)	162.447 ± 38.336	210.768 ± 44.559^a^	0.034
LDL (mmol/L)	1.448 ± 0.286	1.807 ± 0.378^a^	0.048
HDL (mmol/L)	1.828 ± 0.276	1.532 ± 0.205^a^	0.027

^a^ Values within a row with different superscripts differ significantly at *p* < 0.05.

### 3.3 Metagenomics Sequencing Data

Rumen fluid samples (*n* = 18) from HIMF and LIMF Pingliang Red steers were sequenced. After quality control, 180.76 Gb of clean data were produced (average 10.04 Gb/sample, mean GC 50.04%; Table S1). Assembly generated 3,122,268 contigs (N50 = 1727 bp, N90 = 506 bp). Following CDS prediction and CD‐HIT clustering, a non‐redundant gene catalog comprising 2,264,551 genes was constructed (Tables S2 and S3).

### 3.4 Characterization of the Phylogenetic Compositions of the Rumen Microbiome

The rumen microbiota comprised 98.79% Bacteria, 0.91% Archaea, 0.29% Eukaryota, and 0.01% Viruses (Table S4). *Firmicutes* (*Bacillota*) and *Bacteroidota* were the dominant phyla in both groups (Table S5). At the phylum level, 70 phyla were identified; the five most abundant were *Bacteroidetes* (45.72% ± 6.13%), *Firmicutes* (21.78% ± 4.06%), *Proteobacteria* (15.21% ± 1.94%), *Actinomycetota* (6.31% ± 0.59%), and *Fibrobacterota* (3.54% ± 2.00%). The only archaeal phylum detected was *Euryarchaeota* (0.89% ± 0.22%), and *Apicomplexa* (0.07% ± 0.01%) was the top‐ranked eukaryotic phylum (Table S5).

A total of 2232 microbial genera were identified. The five most abundant genera were *Prevotella* (18.75% ± 3.64%), *Xylanibacter* (7.46% ± 1.39%), *Aristaeella* (4.96% ± 2.67%), *Bacteroides* (3.84% ± 0.38%), and *Fibrobacter* (3.62% ± 1.92%) (Table S6). *Methanobrevibacter* (0.65% ± 0.19%) was the only archaeal genus in the top 30. Of 8884 species detected, 8363 were shared across all 18 rumen samples, 284 were unique to the HIMF group, and 237 were specific to the LIMF group (Figure S1).

### 3.5 Microbial Diversity Analysis

**FIGURE 2:**
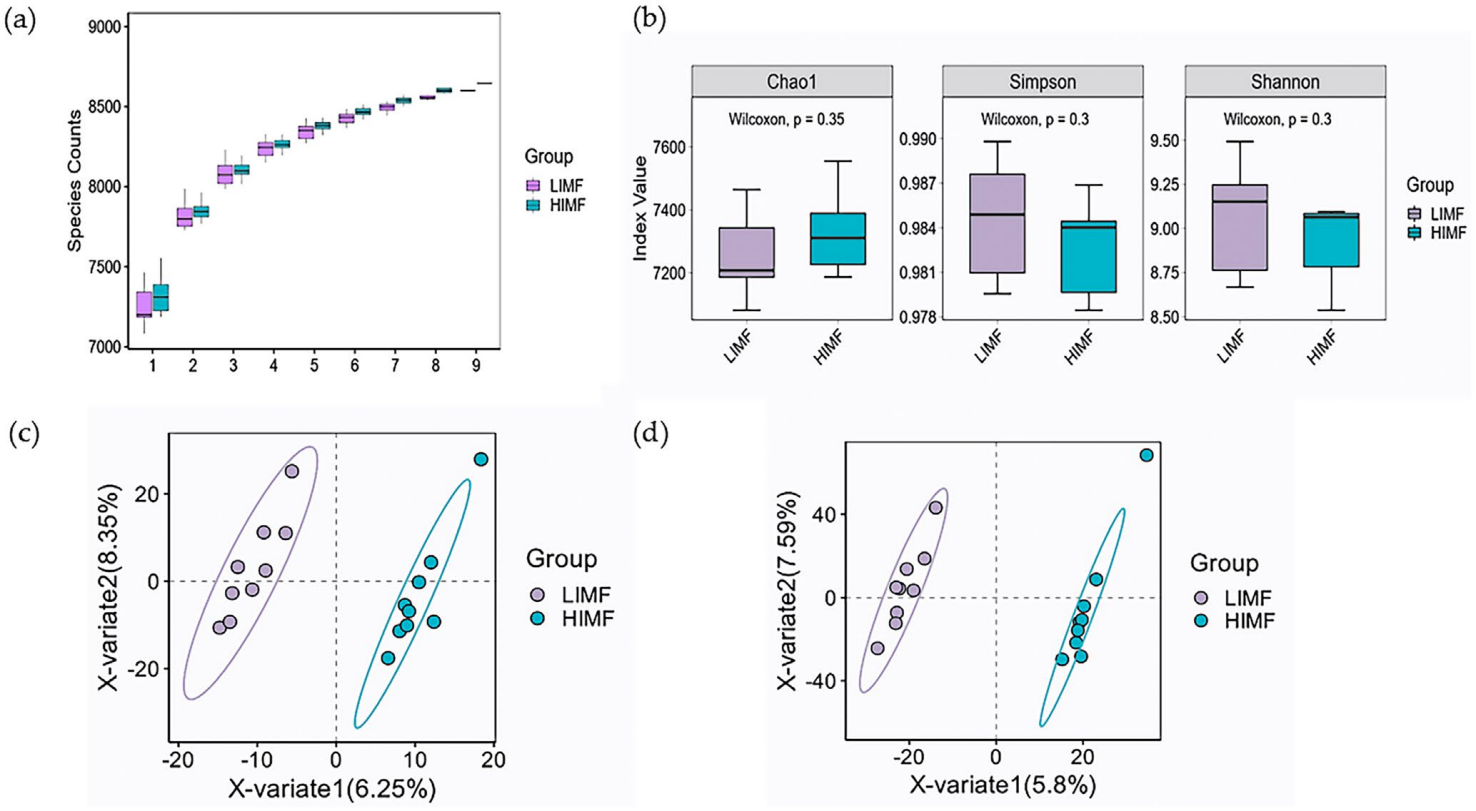
Microbial alpha diversity analysis between the HIMF and LIMF groups. (a) Box plot of the dilution curve, with the *x*‐axis representing the number of samples and the *y*‐axis representing the number of detected species. (b) Box plot of α‐diversity indices. (c) PLS‐DA at the genus level. (d) PLS‐DA at the species level.

**FIGURE 3:**
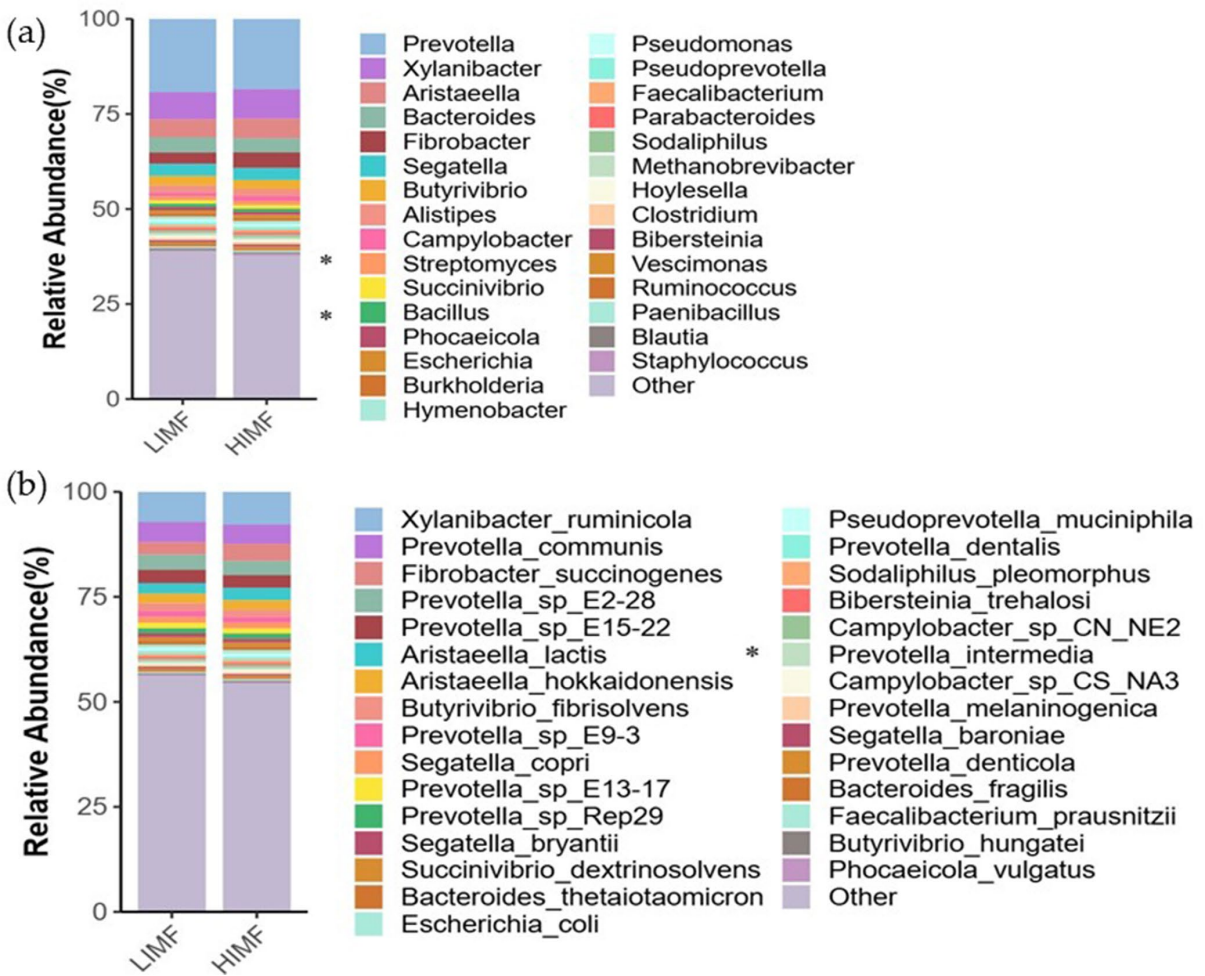
Microbial composition in rumen between the HIMF and LIMF groups. (a) Differential microbial composition at the genus level; (b) Differential microbial composition at the species level.

Figure 2a showed that the species accumulation curve plateaued and confirmed sufficient sampling. No significant differences were observed in α‐ or β‐diversity between the HIMF and LIMF groups (*p* > 0.05, Figure 2b). At the genus level, *Prevotella* (18.47% ± 4.15%), *Xylanibacter* (7.77% ± 1.38%), *Aristaeella* (5.18% ± 3.25%), and *Bacteroides* (3.71% ± 0.38%) were most abundant in the HIMF group. Among the top 30 genera (Figure 3a), *Streptomyces* was significantly enriched, whereas *Bacillus* was depleted in the HIMF group (*p* < 0.05). Additionally, *Prevotella intermedia* was significantly more abundant in the HIMF group (Figure 3b, *p* < 0.05). The enrichment of *Prevotella intermedia* and *Streptomyces*, coupled with reduced *Bacillus* abundance, characterized distinct rumen microbiota of HIMF cattle.

### 3.6 Functional Analysis Based on CAZy and KEGG

**FIGURE 4:**
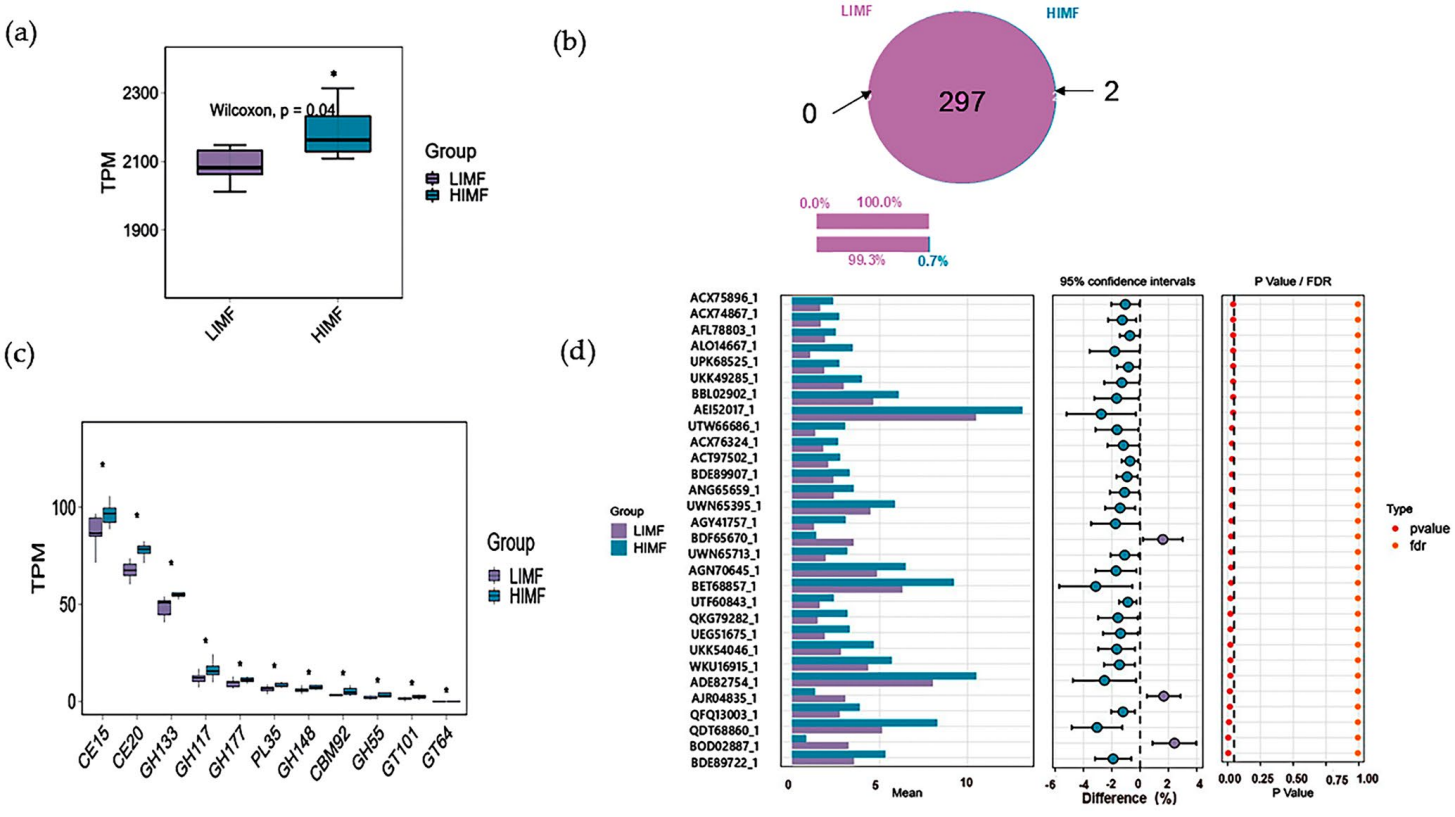
Analysis of rumen microbial function in HIMF and LIMF groups. (a) CAZy functional analysis, (b) Venn diagram of level 2 differences, (c) functional differences at level 2, and (d) STAMP analysis at level 3 based on the CAZy database.

**FIGURE 5:**
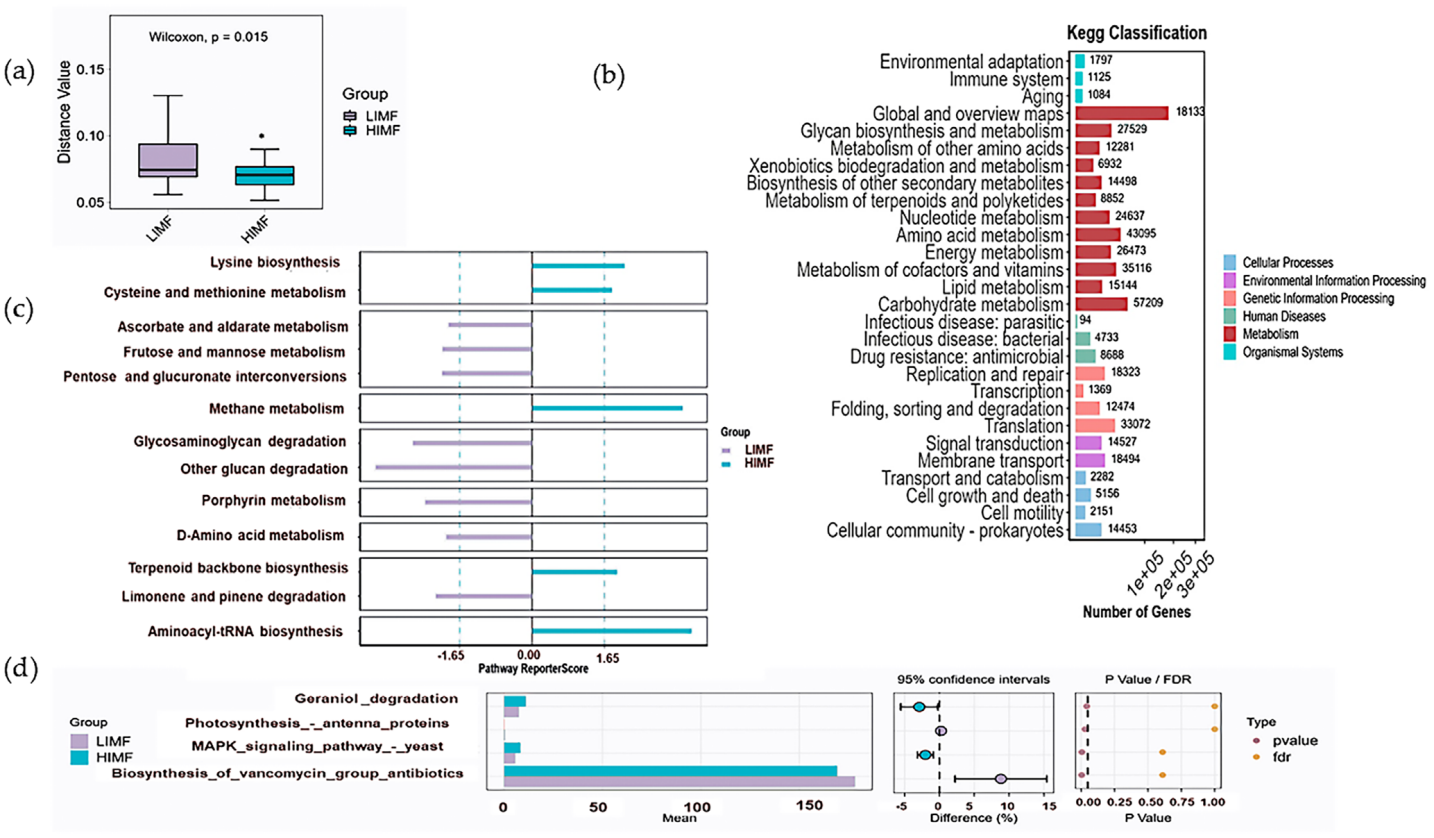
Functional β‐diversity analysis of KEGG pathways. (a) Box plot of KEGG β‐diversity function. (b) Bar chart of functional gene statistics. (c) Functional KEGG pathway enrichment map. (d) STAMP analysis results for HIMF and LIMF groups.

Microbial genes (2,264,551) were annotated against CAZy and KEGG databases, yielding 60,409 CAZyme‐encoding genes (2.74%). CAZyme profiles differed significantly between groups (Figure 4a). GT128 and GT133 were detected exclusively in the HIMF group, which also showed enrichment of GHs (GH55, GH117, GH133, GH148, GH177), CEs (CE15, CE20), PL35, CBM92, and GTs (GT64, GT101) (*p* < 0.05, Figure 4b,c). CBM48 (BET68857.1) abundance was higher in HIMF at hierarchy level 3 (*p* < 0.05, Figure 4d). KEGG pathways also differed between groups (*p* < 0.05, Figure 5a). Functional genes were primarily associated with cellular processes and environmental and genetic information processing (Figure 5b). Among 15 differential pathways, lysine and sulfur amino acid metabolism and the yeast MAPK signaling pathway were enriched in HIMF, whereas streptomycin biosynthesis was enriched in LIMF (Figure 5c,d).

### 3.7 Associations Between Rumen Microbiota and IMF

**FIGURE 6:**
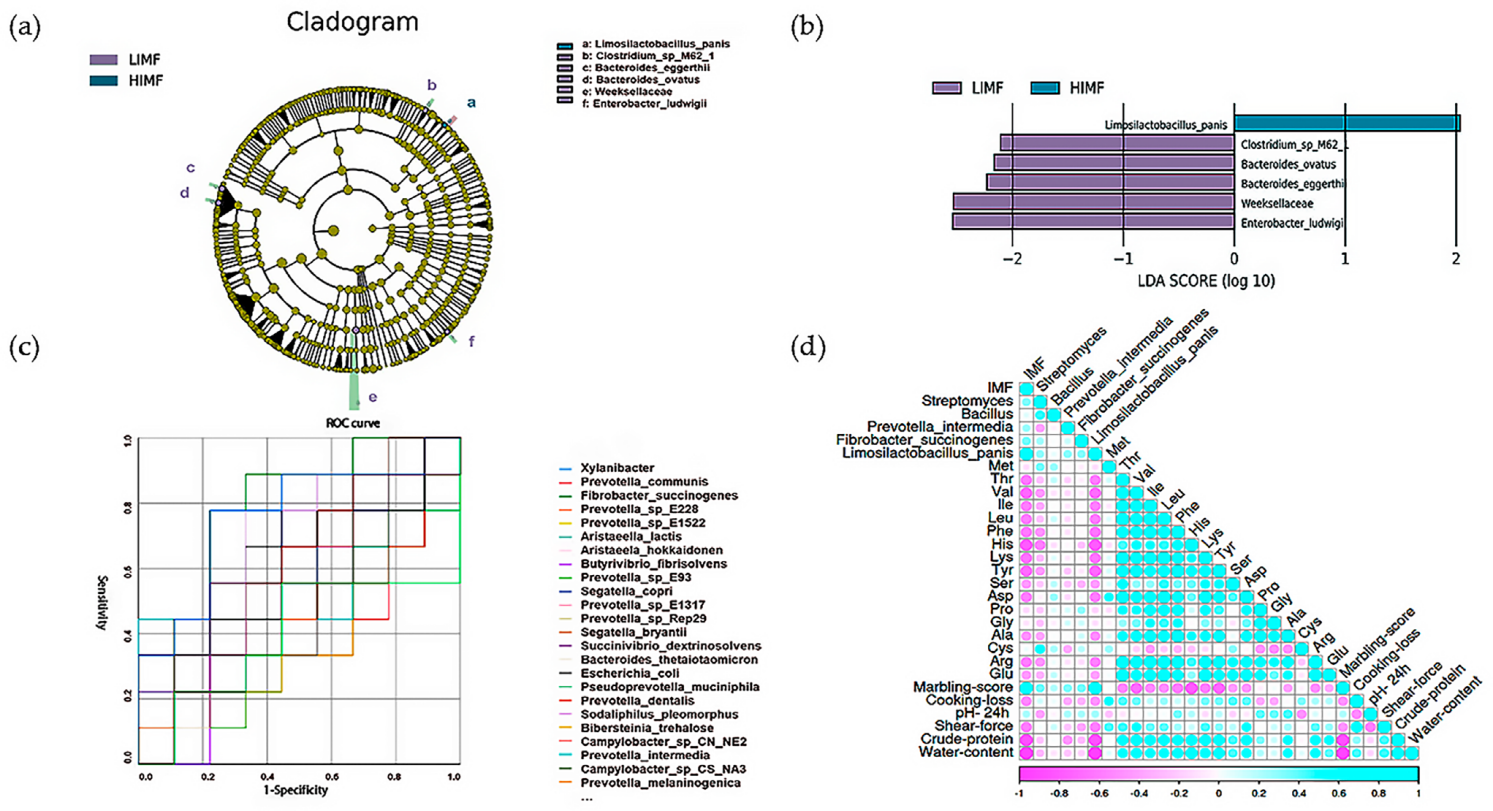
Identification of HIMF biomarkers and their association with meat quality. (a) LEfSe circular evolutionary branching diagram. (b) LEfSe LDA value distribution bar chart (classifications with LDA > 2 are shown). (c) ROC curve of bacterial strains and high intramuscular fat. The *x*‐axis represents 1‐specificity, and the *y*‐axis represents sensitivity; the area under the curve (AUC) is indicated. (d) Heatmap of correlations among IMF, microorganisms, and meat quality.

Correlations between rumen microbiota and fat deposition traits (IMF% and marbling) were analyzed in Pingliang Red cattle. LEfSe analysis identified *Limosilactobacillus panis* as the primary discriminator between HIMF and LIMF groups (Figure 6a,b). ROC analysis revealed moderate predictive power (Liu and Duan 2022) for both *Limosilactobacillus panis* (AUC = 0.765) and *Fibrobacter succinogenes* (AUC = 0.753) (Figure 6c; Table S8). Spearman correlation analysis showed that IMF% was negatively correlated with crude protein, shear force, moisture, and several amino acids (*p* < 0.05), whereas it was positively correlated with *Limosilactobacillus panis* abundance (*p* < 0.05, Figure 6d).

### 3.8 Metabolic Pathway for Pingliang Red Cattle in HIMF Group

**FIGURE 7:**
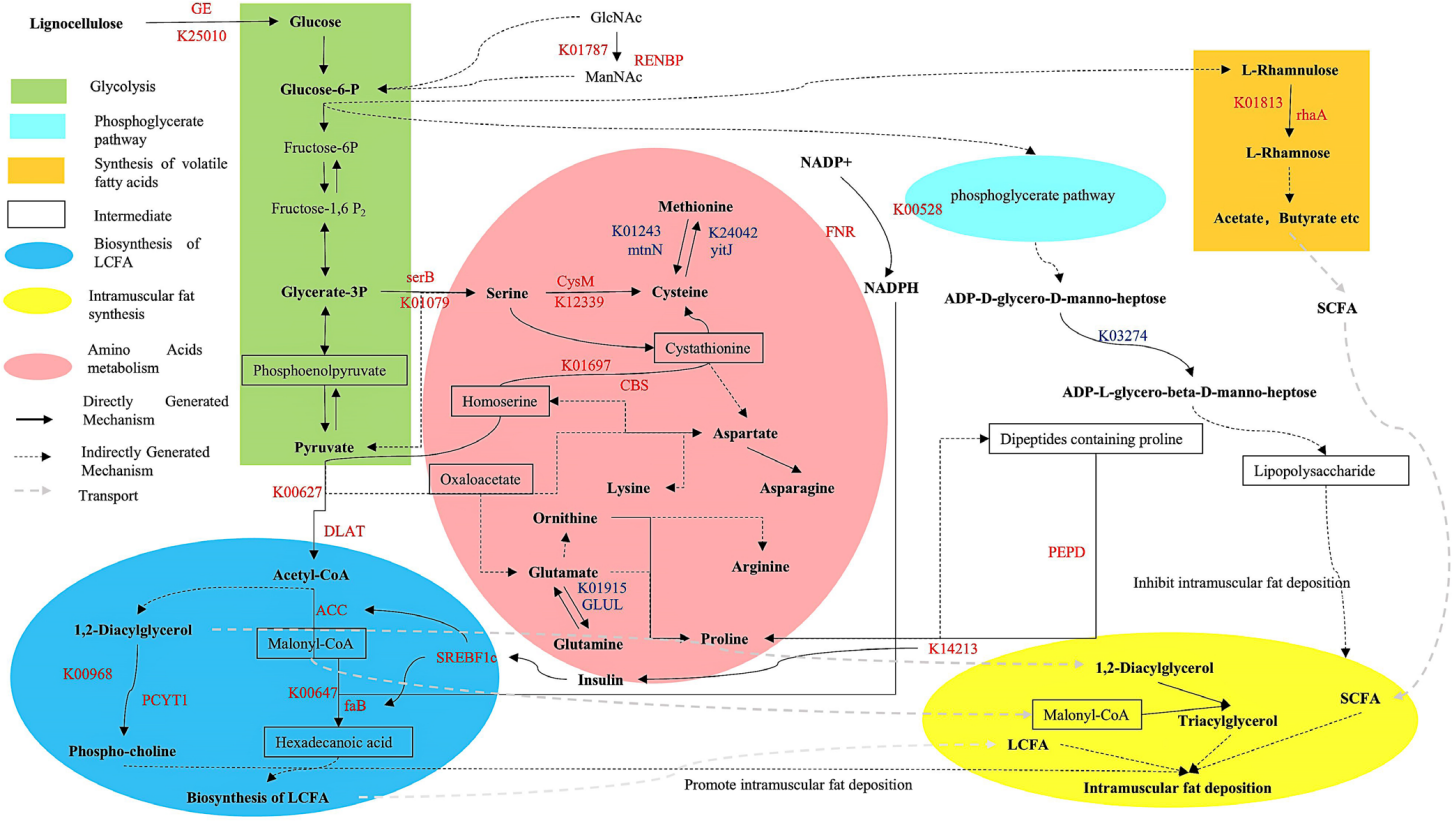
Pathways of IMF generation regulated by microbial genes. Comparison of relative abundances of KO enzymes related to metabolic pathways between the HIMF and LIMF groups (Mann–Whitney *U* test, *n* = 5 per group; Table S9). Enzymes in red indicate up‐regulation, while those in blue indicate down‐regulation.

As illustrated in Figure 7, HIMF cattle exhibited upregulated glucuronoyl esterase (GE), RENBP, and ferredoxin‐NADP^+^ reductase (FNR), which enhanced glycolysis and pentose phosphate pathway fluxes, thereby increasing NADPH supply for long‐chain fatty acid (LCFA) synthesis. Elevated Xaa‐Pro dipeptidase (PEPD) activity preserved insulin sensitivity, subsequently modulating lipogenic enzyme and LCFA synthesis. Concurrently, downregulation of lipopolysaccharide biosynthesis (K03274) decreased lipopolysaccharides precursor production and modulated IMF deposition. Glucose‐6‐phosphate was converted to SCFAs via rhaA to promote IMF deposition; pyruvate was converted to acetyl‐CoA and subsequently utilized for fatty acid elongation and triacylglycerol (TAG) synthesis. Enhanced phosphatidylcholine production via PCYT1 further supported lipid droplet formation, collectively promoting IMF accumulation.

## 4 Discussion

### 4.1 Meat Quality and Fatty Acid Composition

Beef marbling is a primary indicator of meat quality, closely associated with tenderness, flavor, and juiciness (Erena et al. 2024). In this study, 18 Pingliang Red cattle were stratified into high (HIMF) and low (LIMF) intramuscular fat groups. The HIMF group exhibited significantly higher IMF% (12.7% ± 2.4%) and marbling scores (4.7 ± 0.5) compared with LIMF (3.9% ± 0.9% and 2.8 ± 0.4, respectively), alongside improved tenderness (Wang et al. 2025). HIMF beef had lower protein and amino acid contents but higher total fatty acids, particularly unsaturated fatty acids including C20:2 and C18:1n‐9c. Conversely, saturated fatty acids (e.g., C16:0) were reduced. Elevated n‐6 and n‐3 polyunsaturated fatty acids in HIMF may benefit human health by alleviating hyperuricemia (Mehrban et al. 2021). Additionally, higher contents of flavor‐related medium‐chain fatty acids (C10:0, C12:0) in HIMF could enhance volatile compound formation during heating, contributing to intensified beef flavor (Park et al. 2022).

### 4.2 Rumen Microbiome Structure and IMF Deposition

Metagenomic sequencing characterized rumen microbial composition and functional capacity in this study. Rumen microbiota structure significantly influences beef marbling, independent of genetic and nutritional factors (Holman et al. 2024; Yang, Chen, et al. 2024). We found that *Firmicutes* (*Bacillota*) and *Bacteroidota* dominated the rumen microbiota of both LIMF and HIMF groups (Table S5), supporting their linkage to IMF% (Magne et al. 2020). This dominance aligns with Zhou et al. (2023), who identified *Bacteroidetes* and *Firmicutes* as core phyla in Qinchuan cattle rumen and feces. Our findings contrast with previous reports on microbial diversity and IMF relationships. Yang, Chen, et al. (2024) demonstrated that rumen microbiome composition correlated with IMF% in Xiangzhong Black cattle. Shi et al. (2024) further reported higher gastrointestinal microbial diversity in high‐marbling cattle, characterized by increased *Firmicutes* and *Akkermansia* but decreased *Bacteroidetes* and *Prevotella*. Conversely, Holman et al. (2024) reported positive correlations between *Prevotella* and IMF in Angus × Simmental steers. Similarly, Chen, Sun, et al. (2022) found that Angus cattle had higher IMF content than Xinjiang Brown cattle, with IMF positively correlating with *Prevotella*, *rumen cocci*, and succinic acid. This discrepancy in *Prevotella*‐IMF relationships may reflect differences in sample origin, community complexity, and environment, warranting further investigation. Moreover, previous work indicated higher ASV richness and Chao1 diversity in high‐marbling cattle (Kim et al. 2020). In contrast, we detected no α‐ or β‐diversity differences between HIMF and LIMF groups, possibly due to breed, grouping criteria, and sample size variations. Nevertheless, the correlations of rumen microbiome with host IMF% were investigated in this study. The results provided a reference for improving cattle production performance by regulating the rumen microbiome.

### 4.3 Microbial Functional Capacity and IMF Deposition

CAZyme activities were significantly higher in the HIMF group, manifested as enhanced degradation of cellulose and hemicellulose. This is consistent with Sato et al. (2021), who reported enriched genes for cellulose and hemicellulose degradation and abundant *fibrolytic bacteria* ( *Fibrobacter succinogenes* , *Ruminococcus*, *Treponema*) in high‐marbling Japanese Brown cattle. *Fibrobacter succinogenes* , a dominant cellulolytic species in the rumen ecosystem (Ogawa et al. 2025), degrades plant cell wall cellulose into *fermentable* sugars (Yeoman et al. 2021). The resulting metabolites, succinate and acetate, enhance rumen metabolic activity (Jun et al. 2007) and provide carbon substrates for host lipogenesis.

Beyond fiber degradation, *Limosilactobacillus panis* (AUC = 0.765) and *Fibrobacter succinogenes* (AUC = 0.753) emerged as potential IMF regulators in Pingliang Red cattle, with *Limosilactobacillus panis* showing significant positive correlation with IMF content. Although direct evidence for *Limosilactobacillus panis* in lipid metabolism is limited, related *Lactobacillus* species regulate host energy homeostasis. *Limosilactobacillus plantarum* KC28 exerts anti‐obesity effects in high‐fat diet‐fed mice (Huang et al. 2020), and *Limosilactobacillus sakei* ADM14 reshapes gut microbiota and mitigates diet‐induced obesity (Won et al. 2020). *Limosilactobacillus reuteri* promotes *Bacteroides acidifaciens* enrichment, which synthesizes pentadecanoic acid (C15:0), thereby inhibiting pro‐inflammatory cytokine production and alleviating intestinal inflammation (Abuqwider et al. 2022; Jiang et al. 2023). These findings suggest that *L. panis* may contribute to IMF deposition through host‐microbe metabolic interactions, though the underlying mechanisms warrant further investigation.

### 4.4 Volatile Fatty Acids and Host Lipid Metabolism

Ruminal acetate concentration was higher in the HIMF group, indicating enhanced supply to muscle tissue. This suggests that increased ruminal cellulose degradation promotes IMF deposition (Fang and Richardson 2005). Acetate produced via microbial degradation of cellulose and hemicellulose is primarily utilized by peripheral tissues, especially adipose and muscle tissues (Bergman 1990), serving as a primary substrate for de novo fatty acid synthesis (Xiong et al. 2024).

We acknowledge that direct tracking of fatty acid metabolism (e.g., isotope labeling) was not performed. However, integration of metagenomic and VFAs data supports that HIMF microbiota enhanced VFAs production. Serum biochemical indices further demonstrate that VFAs regulate IMF deposition through modulation of systemic metabolic parameters (Pan et al. 2025).

VFAs mediate various physiological processes and regulate host energy metabolism (Urga et al. 2025). Saturated and monounsaturated fatty acids are synthesized via de novo lipogenesis using VFAs and glucose, whereas polyunsaturated fatty acids are derived from dietary linoleic and linolenic acids (Xiong et al. 2024). In the context of IMF deposition, VFAs modulate adipocyte differentiation, triglyceride synthesis, angiogenesis, and de novo lipogenesis (Shah et al. 2021).

### 4.5 Amino Acid Metabolism and MAPK Signaling Pathway

The MAPK signaling pathway regulates adipocyte differentiation and fat deposition through ERK, p38 MAPK, and JNK cascades (Xiang et al. 2024). In chickens, MAPK regulates lipogenesis via the PPAR pathway (Zhu et al. 2010), and its inhibition suppresses lipogenesis in bovine preadipocytes (Li et al. 2023). Sulfur amino acid metabolism also modulates adipogenesis. Cysteine (Cys) and methionine (Met) are essential for porcine adipocyte differentiation (Castellano et al. 2016). Severe Met deficiency impairs proliferation and differentiation, while mild deficiency affects differentiation only. Met and Cys modulate adipocyte differentiation and lipid metabolism through key transcription factors including PPARγ and C/EBPα (Inthanon et al. 2025).

In this study, enhanced ruminal amino acid metabolism in the HIMF group likely accounted for lower Met and Cys contents in the longissimus dorsi muscle. We speculate that the HIMF group utilizes more Met and Cys to support adipocyte differentiation and lipid metabolism, resulting in reduced muscle tissue reserves. The significant enrichment of MAPK signaling in the HIMF group suggests that this pathway is linked to IMF deposition in Pingliang Red cattle, integrating microbial metabolic outputs with host adipogenic programs.

## 5 Conclusion

This study revealed that rumen microbiota significantly influence IMF deposition in Pingliang Red cattle through taxonomic and functional mechanisms. *Limosilactobacillus panis* and *Fibrobacter succinogenes* were identified as potential biomarkers for high IMF content. Functionally, enhanced carbohydrate‐active enzyme activities and elevated volatile fatty acid production in HIMF cattle systematically promoted IMF deposition by coordinating glycolysis, the pentose phosphate pathway, and insulin signaling, thereby increasing NADPH supply and redirecting carbon flux toward fatty acid and triacylglycerol synthesis. These findings provide a foundation for microbiome‐based strategies to improve beef marbling.

## Author Contributions

**Hong Meng:** data curation (equal), methodology (equal), writing – original draft (lead). **Shuanping Zhao:** conceptualization (equal), data curation (equal), methodology (equal), visualization (equal), writing – review and editing (equal). **Hai Jin:** data curation (equal), software (equal), visualization (equal). **Huibin Zhang:** data curation (equal), methodology (equal), software (equal). **Qian Li:** data curation (equal), methodology (equal), software (equal). **Lupei Zhang:** data curation (equal), software (equal). **Junwei Hu:** data curation (equal), resources (equal). **Fanyun Kong:** investigation (equal), resources (equal). **Xinyi Du:** data curation (equal), project administration (equal). **Qinggang Li:** project administration (equal), software (equal). **Muhammad Ajwad Rahim:** data curation (equal), writing – review and editing (equal). **Lei Xu:** funding acquisition (equal), project administration (equal), supervision (equal), writing – review and editing (equal). **Yanfeng Xue:** conceptualization (equal), funding acquisition (equal), methodology (equal), writing – review and editing (equal).

## Funding

This work was supported by China Agriculture Research System of MOF and MARA, CARS–37. AAU Introduction of High‐level Talent Funds, RC392107. The Project of Beef Cattle Revitalization of Anhui Provincial Science and Technology Department, 202513b10050018. Anhui Provincial Science and Technology Innovation Breakthrough Project (Major Project), 202423m10050001. National Key Research and Development Program of China, 2022YFD1301102.

## Conflicts of Interest

The authors declare no conflicts of interest.

## Supporting information


**Figure S1:** Venn/UpSetR of all 18 samples at species level.
**Table S1:** Clean sequence reads obtained from all the 18 samples in the HIMF and LIMF groups.
**Table S2:** Assembly statistics of metagenomes from 18 rumen fluid samples.
**Table S3:** Gene lenth distribution of all 18 samples.
**Table S4:** Kingdom relative abundance of all 18 samples.
**Table S5:** Top 30 Phylum absolute abundance of 18 samples.
**Table S6:** Top 30 Genus relative abundance of 18 samples.
**Table S7:** non‐redundant microbial genes in different databases of all samples.
**Table S8:** The AUC values of microbiota.
**Table S9:** Comparisons of the relative abundance of KO enzymes.

## Data Availability

The data that support the findings of this study are available on request from the corresponding author. The data are not publicly available due to privacy or ethical restrictions.
