# Supplementary material for: Unveiling the Role of Rumen Microbiome in Modulating Intramuscular Fat Deposition of Pingliang Red Cattle
Source: Food Sci Nutr. 2026 Apr 15;14(4):e71681. doi: 10.1002/fsn3.71681 (PMC13080503; doi:10.1002/fsn3.71681)
Supplement: Supplementary file 1 — Figure S1: Venn/UpSetR of all 18 samples at species level. Table S1: Clean sequence reads obtained from all the 18 samples in the HIMF and LIMF groups. Table S2: Assembly statistics of metagenomes from 18 rumen fluid samples. Table S3: Gene lenth distribution of all 18 samples. Table S4: Kingdom relative abundance of all 18 samples. Table S5: Top 30 Phylum absolute abundance of 18 samples. Table S6: Top 30 Genus relative abundance of 18 samples. Table S7: non‐redundant microbial genes in different databases of all samples. Table S8: The AUC values of microbiota. Table S9: Comparisons of the relative abundance of KO enzymes. [file FSN3-14-e71681-s001.docx]

**Supplementary Fig S1 Venn/UpSetR of all 18 samples at species level**


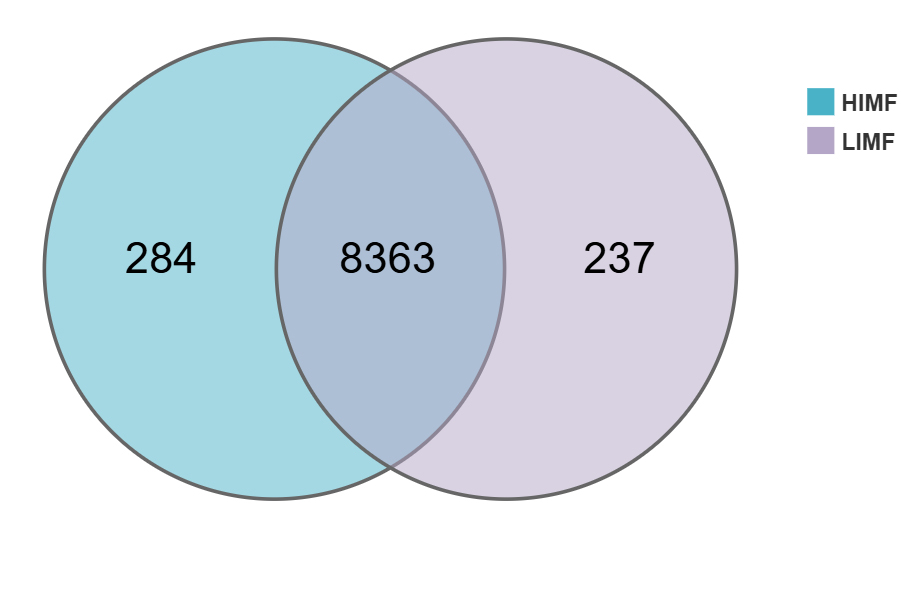


**Supplementary Table S1 Clean sequence reads obtained from all the 18 samples in the HIMF and LIMF groups**

| Host Rate (%) | Clean Bases (Remove Host) | Clean Data Rate (%) | Clean Reads (Remove Host) | GC (%) | Q30 (%) | Q20 (%) | Clean Bases | Clean Reads | Raw Bases | Raw Reads | Sample |
| --- | --- | --- | --- | --- | --- | --- | --- | --- | --- | --- | --- |
| 0.25 | 10001040300.00 | 94.94 | 66673602.00 | 50.00 | 95.52 | 99.00 | 10025591700.00 | 66837278.00 | 10560000000.00 | 70400000.00 | H |
| 0.19 | 10047122100.00 | 95.32 | 66980814.00 | 49.60 | 95.10 | 98.80 | 10065923400.00 | 67106156.00 | 10560000000.00 | 70400000.00 | H |
| 0.43 | 10021288200.00 | 94.77 | 66808588.00 | 50.00 | 94.16 | 98.00 | 10064466000.00 | 67096440.00 | 10620000000.00 | 70800000.00 | H |
| 0.20 | 10031949600.00 | 95.30 | 66879664.00 | 50.80 | 94.79 | 98.61 | 10052104800.00 | 67014032.00 | 10548000000.00 | 70320000.00 | H |
| 0.15 | 10049470200.00 | 94.99 | 66996468.00 | 50.00 | 94.80 | 99.00 | 10064827200.00 | 67098848.00 | 10596000000.00 | 70640000.00 | H |
| 0.69 | 9947577300.00 | 95.18 | 66317182.00 | 51.06 | 95.21 | 98.77 | 10016415900.00 | 66776106.00 | 10524000000.00 | 70160000.00 | H |
| 0.26 | 9993299700.00 | 95.21 | 66621998.00 | 49.00 | 95.24 | 99.00 | 10019451600.00 | 66796344.00 | 10524000000.00 | 70160000.00 | H |
| 0.48 | 9985314000.00 | 95.12 | 66568760.00 | 49.91 | 94.94 | 98.77 | 10033542600.00 | 66890284.00 | 10548000000.00 | 70320000.00 | H |
| 0.14 | 10003999200.00 | 95.30 | 66693328.00 | 50.00 | 93.56 | 99.00 | 10018231800.00 | 66788212.00 | 10512000000.00 | 70080000.00 | H |
| 0.16 | 10032750300.00 | 95.27 | 66885002.00 | 49.26 | 94.28 | 98.60 | 10048862100.00 | 66992414.00 | 10548000000.00 | 70320000.00 | L |
| 0.37 | 10029502200.00 | 97.66 | 66863348.00 | 51.00 | 94.06 | 99.00 | 10067128800.00 | 67114192.00 | 10308000000.00 | 68720000.00 | L |
| 0.04 | 10050396300.00 | 98.11 | 67002642.00 | 49.11 | 93.61 | 98.35 | 10054239900.00 | 67028266.00 | 10248000000.00 | 68320000.00 | L |
| 0.11 | 10032103200.00 | 95.11 | 66880688.00 | 49.00 | 95.25 | 99.00 | 10043393400.00 | 66955956.00 | 10560000000.00 | 70400000.00 | L |
| 0.22 | 10043365500.00 | 97.42 | 66955770.00 | 50.33 | 94.28 | 98.55 | 10065300900.00 | 67102006.00 | 10332000000.00 | 68880000.00 | L |
| 0.28 | 10010789400.00 | 98.19 | 66738596.00 | 49.00 | 94.26 | 99.00 | 10038603300.00 | 66924022.00 | 10224000000.00 | 68160000.00 | L |
| 0.27 | 9990963300.00 | 97.64 | 66606422.00 | 49.95 | 93.72 | 98.40 | 10017459600.00 | 66783064.00 | 10260000000.00 | 68400000.00 | L |
| 0.16 | 10028314200.00 | 97.90 | 66855428.00 | 51.00 | 94.17 | 99.00 | 10044363300.00 | 66962422.00 | 10260000000.00 | 68400000.00 | L |
| 0.13 | 10004251500.00 | 97.64 | 66695010.00 | 50.46 | 94.03 | 98.59 | 10017702600.00 | 66784684.00 | 10260000000.00 | 68400000.00 | L |
| 0.25 | 10016860917.00 | 96.17 | 66779072.78 | 50.04 | 94.50 | 98.64 | 10042089383.00 | 66947262.56 | 10444000000.00 | 69626666.67 | mean |
| 4.52 | 180303496500.00 | 1731.07 | 1202023310.00 | 901.00 | 1700.98 | 1776.00 | 180757608900.00 | 1205050726.00 | 187992000000.00 | 1253280000.00 | total |

**Supplementary Table S2 Assembly statistics of metagenomes from 18 rumen fluid samples**

| De-duplicated CDS Number | Prediced CDS Number | average_size | min | max | n90 | n50 | assembly_length | contig_number | software |
| --- | --- | --- | --- | --- | --- | --- | --- | --- | --- |
| 299726.00 | 361503.00 | 1185.00 | 300.00 | 421174.00 | 508.00 | 1617.00 | 227411537.00 | 191881.00 | Megahit |
| 275733.00 | 333655.00 | 1272.00 | 300.00 | 420040.00 | 542.00 | 1840.00 | 215177093.00 | 169122.00 | Megahit |
| 243108.00 | 295999.00 | 1144.00 | 300.00 | 371383.00 | 502.00 | 1442.00 | 181518509.00 | 158610.00 | Megahit |
| 274454.00 | 339765.00 | 1256.00 | 300.00 | 598319.00 | 495.00 | 1852.00 | 219692045.00 | 174875.00 | Megahit |
| 325644.00 | 391252.00 | 1247.00 | 300.00 | 272422.00 | 540.00 | 1807.00 | 251603209.00 | 201697.00 | Megahit |
| 285359.00 | 348804.00 | 1172.00 | 300.00 | 324158.00 | 499.00 | 1586.00 | 216882474.00 | 184967.00 | Megahit |
| 269704.00 | 333104.00 | 1196.00 | 300.00 | 478409.00 | 505.00 | 1736.00 | 210066581.00 | 175604.00 | Megahit |
| 276625.00 | 347566.00 | 1072.00 | 300.00 | 380596.00 | 468.00 | 1351.00 | 207792269.00 | 193774.00 | Megahit |
| 268635.00 | 323042.00 | 1327.00 | 300.00 | 439233.00 | 548.00 | 2105.00 | 214752571.00 | 161736.00 | Megahit |
| 280206.00 | 340749.00 | 1191.00 | 300.00 | 520126.00 | 512.00 | 1614.00 | 213122858.00 | 178834.00 | Megahit |
| 272728.00 | 339692.00 | 1125.00 | 300.00 | 319102.00 | 480.00 | 1517.00 | 207614616.00 | 184407.00 | Megahit |
| 191907.00 | 222251.00 | 860.00 | 300.00 | 170176.00 | 357.00 | 1025.00 | 117588082.00 | 136703.00 | Megahit |
| 273328.00 | 327339.00 | 1383.00 | 300.00 | 478334.00 | 565.00 | 2348.00 | 221422521.00 | 160091.00 | Megahit |
| 267644.00 | 331133.00 | 1229.00 | 300.00 | 477662.00 | 514.00 | 1792.00 | 210564529.00 | 171281.00 | Megahit |
| 249989.00 | 301743.00 | 1255.00 | 300.00 | 608972.00 | 524.00 | 1827.00 | 195115158.00 | 155428.00 | Megahit |
| 301823.00 | 362135.00 | 1241.00 | 300.00 | 378936.00 | 502.00 | 1867.00 | 233245194.00 | 187920.00 | Megahit |
| 274425.00 | 346626.00 | 1277.00 | 300.00 | 341340.00 | 539.00 | 1905.00 | 226452056.00 | 177329.00 | Megahit |
| 246364.00 | 304221.00 | 1232.00 | 300.00 | 768346.00 | 505.00 | 1864.00 | 194701064.00 | 158009.00 | Megahit |
| 4877402.00 | 5950579.00 | 21664.00 | 5400.00 | 7768728.00 | 9105.00 | 31095.00 | 3764722366.00 | 3122268.00 | total |
| 270966.78 | 330587.72 | 1203.56 | 300.00 | 431596.00 | 505.83 | 1727.50 | 209151242.56 | 173459.33 | mean |
| 27119.92228 | 34418.01779 | 109.1050575 | 0 | 133814.9247 | 43.29389 | 284.01179 | 26854144.71 | 15875.10639 | SD |

**Supplementary Table S3 Gene lenth distribution of all 18 samples**

| gene_length | gene_number |
| --- | --- |
| 0~199 | 261365 |
| 200~499 | 950,710 |
| 500~999 | 697905 |
| 1000~1499 | 219,670 |
| 1500~1999 | 72060 |
| 2000~2499 | 32,193 |
| 2500~2999 | 14253 |
| 3000~4999 | 13,722 |
| 5000~9999 | 2287 |
| 10000~14999 | 309 |
| 15000~19999 | 47 |
| 20000~24999 | 20 |
| 25000~30000 | 8 |
| >= 30000 | 2 |

**Supplementary Table S4 Kindom raletive abundance of all 18 samples**

| Bacteria | 98.7846 % |
| --- | --- |
| Archaea | 0.9105 % |
| Eukaryota | 0.2921 % |
| Viruses | 0.0128 % |

**Supplementary Table S5 Top 30 Phylum absolute abundance of 18 samples**

| Bacteroidota | Bacillota | Pseudomonadota | Actinomycetota | Fibrobacterota | Campylobacterota | Thermodesulfobacteriota | Euryarchaeota | Spirochaetota | Planctomycetota | Cyanobacteriota | Verrucomicrobiota | Myxococcota | Mycoplasmatota | Acidobacteriota | Deinococcota | Ascomycota | Synergistota | Chloroflexota | Fusobacteriota | Chlorobiota | Rhodothermota | Apicomplexa | Kiritimatiellota | Thermotogota | Gemmatimonadota | Basidiomycota | Bdellovibrionota | Nitrospirota | Armatimonadota | category |
| --- | --- | --- | --- | --- | --- | --- | --- | --- | --- | --- | --- | --- | --- | --- | --- | --- | --- | --- | --- | --- | --- | --- | --- | --- | --- | --- | --- | --- | --- | --- |
| 938085 | 394929 | 258755 | 125512 | 139888 | 14690 | 19233 | 16308 | 8753 | 8286 | 7700 | 7455 | 6584 | 5656 | 5392 | 3725 | 3255 | 2488 | 1947 | 1845 | 1961 | 1463 | 1173 | 1107 | 667 | 746 | 719 | 611 | 494 | 312 | H |
| 801985 | 395854 | 328608 | 121913 | 101719 | 48560 | 21968 | 24674 | 14369 | 8657 | 9238 | 7276 | 6132 | 6776 | 5542 | 3594 | 3206 | 2822 | 2455 | 2438 | 1826 | 1497 | 2306 | 958 | 822 | 695 | 653 | 718 | 672 | 334 | H |
| 718421 | 525293 | 296405 | 132097 | 101411 | 17987 | 22310 | 12526 | 13557 | 9445 | 8810 | 8167 | 6493 | 6603 | 5116 | 3891 | 2901 | 3263 | 2372 | 2931 | 2464 | 1483 | 1655 | 1287 | 890 | 752 | 640 | 515 | 490 | 394 | H |
| 927889 | 386413 | 289492 | 126645 | 37336 | 41407 | 20978 | 13205 | 8378 | 8442 | 8673 | 7166 | 6570 | 5485 | 5330 | 4211 | 2504 | 3192 | 2578 | 2021 | 1993 | 1561 | 1149 | 968 | 765 | 717 | 650 | 552 | 581 | 421 | H |
| 862820 | 456436 | 299607 | 124656 | 59129 | 29815 | 21139 | 16130 | 14722 | 9293 | 8547 | 8466 | 6603 | 8252 | 5343 | 3752 | 3122 | 2887 | 2218 | 3368 | 1750 | 1282 | 1331 | 1208 | 1050 | 760 | 652 | 748 | 585 | 370 | H |
| 647775 | 598847 | 332186 | 132464 | 48554 | 19959 | 22434 | 11463 | 15416 | 8480 | 8117 | 8001 | 6933 | 3267 | 6200 | 4420 | 2770 | 2822 | 2633 | 2016 | 2031 | 1341 | 950 | 1011 | 622 | 598 | 773 | 485 | 569 | 499 | H |
| 1066409 | 337669 | 262592 | 114034 | 91111 | 10734 | 18218 | 16973 | 8220 | 8378 | 8393 | 7803 | 5915 | 7169 | 4595 | 3407 | 3011 | 2528 | 2164 | 2463 | 1604 | 1204 | 1660 | 968 | 873 | 627 | 603 | 594 | 387 | 458 | H |
| 972667 | 353620 | 260975 | 117500 | 77170 | 22098 | 19976 | 16433 | 12575 | 8440 | 7632 | 7848 | 6073 | 6304 | 5026 | 3477 | 2917 | 2317 | 2080 | 1919 | 1703 | 1247 | 1061 | 1100 | 941 | 688 | 580 | 737 | 496 | 367 | H |
| 969723 | 336857 | 275479 | 123379 | 58728 | 39778 | 19774 | 24876 | 7420 | 8143 | 8359 | 6957 | 6065 | 6659 | 5822 | 3817 | 2806 | 2291 | 2467 | 2224 | 2130 | 1584 | 1044 | 907 | 835 | 665 | 701 | 597 | 443 | 317 | H |
| 839068 | 430042 | 310263 | 115172 | 44892 | 22604 | 19642 | 25333 | 10873 | 7950 | 9248 | 6927 | 6043 | 7352 | 5711 | 3614 | 3143 | 2139 | 2137 | 2345 | 1738 | 1352 | 1732 | 829 | 664 | 798 | 571 | 673 | 613 | 367 | L |
| 806286 | 451938 | 291764 | 139060 | 51719 | 10376 | 23014 | 20678 | 11248 | 10224 | 8165 | 9211 | 7103 | 6716 | 5493 | 3920 | 2843 | 2939 | 2355 | 2113 | 2090 | 1340 | 937 | 1749 | 933 | 883 | 638 | 570 | 524 | 354 | L |
| 1016577 | 268675 | 226271 | 93540 | 21496 | 6519 | 16967 | 15914 | 7915 | 6544 | 7484 | 5831 | 4803 | 4618 | 4337 | 2991 | 2765 | 2043 | 1655 | 1496 | 1665 | 1317 | 1725 | 548 | 746 | 557 | 677 | 543 | 592 | 239 | L |
| 942572 | 411363 | 294386 | 107237 | 41451 | 25343 | 18413 | 17774 | 11945 | 7498 | 7626 | 7016 | 5638 | 5898 | 5086 | 3389 | 2909 | 2448 | 2123 | 2295 | 1756 | 1439 | 1518 | 989 | 732 | 690 | 572 | 622 | 460 | 248 | L |
| 651287 | 536479 | 394758 | 122992 | 37697 | 36937 | 20625 | 15259 | 14959 | 8327 | 7700 | 7457 | 5727 | 6730 | 4917 | 3792 | 2552 | 2868 | 2368 | 2601 | 1681 | 1458 | 921 | 969 | 889 | 663 | 510 | 594 | 496 | 405 | L |
| 952080 | 466554 | 257345 | 104711 | 97095 | 24029 | 19244 | 11451 | 10956 | 8116 | 8351 | 5975 | 5131 | 5989 | 4177 | 3695 | 2879 | 2334 | 2056 | 2126 | 1838 | 1327 | 1173 | 720 | 754 | 553 | 598 | 618 | 451 | 265 | L |
| 835594 | 380518 | 256107 | 110369 | 163807 | 26160 | 18462 | 14125 | 11521 | 7942 | 7761 | 7494 | 5861 | 6353 | 4706 | 3279 | 2639 | 2437 | 2066 | 1710 | 1681 | 1151 | 851 | 1045 | 786 | 703 | 625 | 666 | 482 | 387 | L |
| 777082 | 393258 | 277964 | 121762 | 22705 | 11713 | 20718 | 13721 | 9352 | 8958 | 9201 | 7681 | 6160 | 5499 | 5538 | 4229 | 3144 | 2594 | 2323 | 1623 | 2124 | 1499 | 1275 | 999 | 788 | 732 | 623 | 811 | 403 | 428 | L |
| 900927 | 330204 | 284034 | 123362 | 28242 | 22548 | 21423 | 17853 | 12641 | 9276 | 8698 | 7494 | 6920 | 5261 | 5453 | 3873 | 3159 | 2814 | 2418 | 1800 | 1984 | 1431 | 1262 | 1113 | 811 | 772 | 592 | 562 | 472 | 265 | L |
| 868180.3889 | 414163.8333 | 288721.7222 | 119800.2778 | 68008.33333 | 23958.72222 | 20252.11111 | 16927.55556 | 11378.88889 | 8466.611111 | 8316.833333 | 7456.944444 | 6153 | 6143.722222 | 5210.222222 | 3726.444444 | 2918.055556 | 2623.666667 | 2245.277778 | 2185.222222 | 1889.944444 | 1387.555556 | 1317.944444 | 1026.388889 | 809.3333333 | 699.9444444 | 632.0555556 | 623.1111111 | 511.6666667 | 357.2222222 | mean |

**Supplementary Table S6 Top 30 Genus relative abundance of 18 samples**

| category | Mean | SD | HIMF-mean | HIMF-SD | LIMF-mean | LIMF-SD | independent t-test(p-value) |
| --- | --- | --- | --- | --- | --- | --- | --- |
| Prevotella | 18.86532778 | 3.809520836 | 18.46706667 | 4.150443393 | 19.26359 | 3.3884353 | 0.421 |
| Xylanibacter | 7.456788889 | 1.518920038 | 7.766344444 | 1.379653665 | 7.147233 | 1.5871807 | 0.365 |
| Aristaeella | 4.961272222 | 2.672561676 | 5.178988889 | 3.251621491 | 4.743556 | 1.9019276 | 0.251 |
| Bacteroides | 3.851511111 | 0.389462244 | 3.710022222 | 0.377426546 | 3.993 | 0.3476675 | 0.667 |
| Fibrobacter | 3.539794444 | 2.008854807 | 4.086766667 | 1.529063267 | 2.992822 | 2.2659664 | 0.537 |
| Segatella | 3.1829 | 0.539961923 | 3.1801 | 0.583091009 | 3.1857 | 0.4930588 | 0.377 |
| Butyrivibrio | 2.409361111 | 0.610570901 | 2.344522222 | 0.450380076 | 2.4742 | 0.7309879 | 0.528 |
| Alistipes | 1.8917 | 0.285490319 | 1.867688889 | 0.207550596 | 1.915711 | 0.3446435 | 0.094 |
| Campylobacter | 1.131516667 | 0.580762216 | 1.287733333 | 0.645821126 | 0.9753 | 0.4568121 | 0.173 |
| Streptomyces | 1.039072222 | 0.1295083 | 1.062666667 | 0.073723342 | 1.015478 | 0.1643054 | 0.034^*^ |
| Succinivibrio | 0.925944444 | 0.709472962 | 0.949311111 | 0.705684321 | 0.902578 | 0.7124755 | 0.753 |
| Bacillus | 0.876611111 | 0.241945607 | 0.837722222 | 0.111943922 | 0.9155 | 0.3186208 | 0.015^*^ |
| Phocaeicola | 0.826505556 | 0.112761816 | 0.793322222 | 0.093494537 | 0.859689 | 0.1203618 | 0.593 |
| Escherichia | 0.82305 | 0.283500827 | 0.875177778 | 0.285282748 | 0.770922 | 0.2718908 | 0.755 |
| Burkholderia | 0.775227778 | 0.26455069 | 0.733355556 | 0.239430074 | 0.8171 | 0.2813198 | 0.628 |
| Hymenobacter | 0.771544444 | 0.105948511 | 0.775111111 | 0.097126406 | 0.767978 | 0.1139789 | 0.592 |
| Pseudomonas | 0.758366667 | 0.141504864 | 0.7224 | 0.04298943 | 0.794333 | 0.1887113 | 0.061 |
| Pseudoprevotella | 0.741872222 | 0.221349705 | 0.762088889 | 0.291916188 | 0.721656 | 0.1093567 | 0.158 |
| Faecalibacterium | 0.680422222 | 0.084590987 | 0.6858 | 0.096063359 | 0.675044 | 0.0708891 | 0.333 |
| Parabacteroides | 0.6788 | 0.085372706 | 0.655211111 | 0.078047599 | 0.702389 | 0.0858644 | 0.692 |
| Sodaliphilus | 0.664516667 | 0.177697865 | 0.6851 | 0.209022864 | 0.643933 | 0.1364374 | 0.207 |
| Methanobrevibacter | 0.664194444 | 0.218039534 | 0.641688889 | 0.23994143 | 0.6867 | 0.1910434 | 0.593 |
| Hoylesella | 0.641394444 | 0.097916396 | 0.626677778 | 0.085935839 | 0.656111 | 0.1065698 | 0.686 |
| Clostridium | 0.605444444 | 0.071958987 | 0.608933333 | 0.072323809 | 0.601956 | 0.0714221 | 0.9 |
| Bibersteinia | 0.585061111 | 0.405117797 | 0.569744444 | 0.473137277 | 0.600378 | 0.322355 | 0.456 |
| Vescimonas | 0.555805556 | 0.10844806 | 0.584555556 | 0.119340476 | 0.527056 | 0.0873309 | 0.263 |
| Ruminococcus | 0.501044444 | 0.089896201 | 0.479366667 | 0.069568639 | 0.522722 | 0.101897 | 0.395 |
| Paenibacillus | 0.45795 | 0.039287293 | 0.448733333 | 0.026575344 | 0.467167 | 0.0470196 | 0.281 |
| Blautia | 0.455433333 | 0.083646 | 0.421444444 | 0.059782329 | 0.489422 | 0.0900494 | 0.399 |
| Staphylococcus | 0.431905556 | 0.319125325 | 0.504477778 | 0.334191358 | 0.359333 | 0.2854201 | 0.529 |

**^*^** Values within a row with different superscripts differ significantly at *P* < 0.05.

**Supplementary Table S7 non-redundant microbial genes in different databases of all samples**

| Sample Name | BacMet | Card | Cazy | Cog | Kegg | Nog | SwissProt |
| --- | --- | --- | --- | --- | --- | --- | --- |
| all | 8292 | 474 | 60409 | 614737 | 518990 | 710770 | 292171 |
| Proportion% | 0.376 | 0.021 | 2.739 | 27.869 | 23.528 | 32.222 | 13.245 |

**Supplementary Table S8 The AUC values of microbiota**

| Test result variable | AUC | Standard error ^a^ | Asymptotic significance ^b^ | Asymptotic 95% confidence interval | |
| --- | --- | --- | --- | --- | --- |
|  |  |  |  | lower limit | upper limit |
| Xylanibacter_ruminicola | 0.6790 | 0.1320 | 0.2000 | 0.4210 | 0.9370 |
| Prevotella_communis | 0.4440 | 0.1430 | 0.6910 | 0.1650 | 0.7240 |
| Fibrobacter_succinogenes | 0.7530 | 0.1250 | 0.0700 | 0.5070 | 0.9990 |
| Prevotella_sp_E228 | 0.4940 | 0.1450 | 0.9650 | 0.2100 | 0.7780 |
| Prevotella_sp_E1522 | 0.5190 | 0.1460 | 0.8950 | 0.2330 | 0.8040 |
| Aristaeella_lactis | 0.5430 | 0.1410 | 0.7570 | 0.2670 | 0.8190 |
| Aristaeella_hokkaidonensis | 0.4940 | 0.1410 | 0.9650 | 0.2170 | 0.7710 |
| Butyrivibrio_fibrisolvens | 0.4690 | 0.1420 | 0.8250 | 0.1900 | 0.7480 |
| Prevotella_sp_E93 | 0.4200 | 0.1400 | 0.5660 | 0.1450 | 0.6950 |
| Segatella_copri | 0.5930 | 0.1460 | 0.5080 | 0.3060 | 0.8790 |
| Prevotella_sp_E1317 | 0.4810 | 0.1430 | 0.8950 | 0.2010 | 0.7620 |
| Prevotella_sp_Rep29 | 0.5680 | 0.1450 | 0.6270 | 0.2830 | 0.8530 |
| Segatella_bryantii | 0.4940 | 0.1490 | 0.9650 | 0.2020 | 0.7850 |
| Succinivibrio_dextrinosolvens | 0.5190 | 0.1430 | 0.8950 | 0.2380 | 0.7990 |
| Bacteroides_thetaiotaomicron | 0.4810 | 0.1440 | 0.8950 | 0.2000 | 0.7630 |
| Escherichia_coli | 0.6420 | 0.1370 | 0.3100 | 0.3740 | 0.9100 |
| Pseudoprevotella_muciniphila | 0.5430 | 0.1450 | 0.7570 | 0.2600 | 0.8270 |
| Prevotella_dentalis | 0.5310 | 0.1430 | 0.8250 | 0.2500 | 0.8120 |
| Sodaliphilus_pleomorphus | 0.5680 | 0.1440 | 0.6270 | 0.2870 | 0.8490 |
| Bibersteinia_trehalosi | 0.4200 | 0.1410 | 0.5660 | 0.1430 | 0.6970 |
| Campylobacter_sp_CN_NE2 | 0.6170 | 0.1400 | 0.4020 | 0.3440 | 0.8910 |
| Prevotella_intermedia | 0.5680 | 0.1480 | 0.6270 | 0.2770 | 0.8580 |
| Campylobacter_sp_CS_NA3 | 0.6170 | 0.1400 | 0.4020 | 0.3440 | 0.8910 |
| Prevotella_melaninogenica | 0.5190 | 0.1470 | 0.8950 | 0.2310 | 0.8060 |
| Segatella_baroniae | 0.6050 | 0.1410 | 0.4530 | 0.3290 | 0.8810 |
| Prevotella_denticola | 0.5190 | 0.1460 | 0.8950 | 0.2330 | 0.8040 |
| Bacteroides_fragilis | 0.5800 | 0.1400 | 0.5660 | 0.3050 | 0.8550 |
| Faecalibacterium_prausnitzii | 0.6790 | 0.1340 | 0.2000 | 0.4170 | 0.9410 |
| Butyrivibrio_hungatei | 0.5680 | 0.1450 | 0.6270 | 0.2850 | 0.8510 |
| Phocaeicola_vulgatus | 0.3950 | 0.1430 | 0.4530 | 0.1150 | 0.6750 |
| Limosilactobacillus_panis | 0.7650 | 0.1180 | 0.0580 | 0.5350 | 0.9960 |

a: Assumed as nonparametric b: Null hypothesis: True region = 0.5

**Supplementary Table S9 Comparisons of the relative abundance of KO enzymes**

| K01243 | K03274 | K24042 | K01915 | K25010 | K01787 | K00528 | K01813 | K14213 | K00627 | K00647 | K00968 | K01079 | K12339 | K01697 |  |
| --- | --- | --- | --- | --- | --- | --- | --- | --- | --- | --- | --- | --- | --- | --- | --- |
| 1444.929 | 401.573 | 46 | 18005.84 | 10739.48 | 1289.23 | 1751.304 | 1881.925 | 3399.969 | 461.702 | 9296.599 | 1258.182 | 2094.782 | 191.193 | 27.251 | H |
| 1404.137 | 423.669 | 78 | 17797.86 | 9970.513 | 1211.974 | 1848.479 | 2267.674 | 3120.567 | 541.668 | 8769.298 | 1291.209 | 2225.986 | 141.742 | 83.791 | H |
| 1499.671 | 359.371 | 31 | 18463.58 | 8913.975 | 1211.721 | 1748.618 | 2018.078 | 3687.804 | 448.219 | 9565.363 | 1663.77 | 2108.516 | 119.384 | 11.999 | H |
| 1506.91 | 402.543 | 52.001 | 18712.09 | 9226.262 | 1283.807 | 2116.678 | 1981.979 | 3237.085 | 431.317 | 9720.629 | 1546.327 | 2445.495 | 130.002 | 43.66 | H |
| 1516.227 | 349.947 | 66.414 | 17720.88 | 9441.81 | 1237.013 | 1703.053 | 1885.626 | 3508.72 | 450.476 | 9920.232 | 1316.989 | 2117.426 | 99.02 | 17 | H |
| 1619.717 | 789.809 | 383.049 | 21745.17 | 6645.314 | 717.972 | 1577.944 | 1467.224 | 2190.552 | 233.517 | 5930.072 | 1057.235 | 1551.093 | 78 | 7 | L |
| 1987.229 | 616.924 | 121.738 | 19464.38 | 7008.803 | 982.996 | 1497.239 | 1601.692 | 2620.966 | 386.838 | 6392.668 | 1064.247 | 1652.712 | 104.393 | 7 | L |
| 1970.257 | 578.398 | 187.64 | 19186.16 | 7123.728 | 1101.871 | 1563.796 | 1562.228 | 2674.866 | 391.99 | 7945.579 | 1020.176 | 1912.102 | 88.564 | 7 | L |
| 1752.064 | 838.259 | 70.008 | 19306 | 7465.364 | 942.44 | 1440.85 | 1592.387 | 2554.196 | 231.71 | 7829.451 | 1016.581 | 1749.364 | 84 | 7.102 | L |
| 1766.997 | 1142.246 | 132.109 | 19001.57 | 6685.381 | 1010.955 | 1218.936 | 1396.913 | 2266.086 | 319.185 | 6669.932 | 1076.768 | 1921.919 | 78.514 | 4 | L |
| 0.009* | 0.009* | 0.016* | 0.009* | 0.009* | 0.009* | 0.009* | 0.009* | 0.009* | 0.009* | 0.009* | 0.009* | 0.009* | 0.016* | 0.008* | Pvalue |

**^*^** Values within a row with different superscripts differ significantly at *P* < 0.05.
